# Tribological Performance of EPDM and TPV Elastomers Against Glass Fiber-Reinforced Polyamide 66 Composites

**DOI:** 10.3390/ma18112515

**Published:** 2025-05-27

**Authors:** Daniel Foltuț, Ion-Dragoș Uțu, Viorel-Aurel Șerban

**Affiliations:** 1 Department of Materials and Manufacturing Engineering, Politehnica University of Timișoara, Mihai Viteazul Blv., 300222 Timisoara, Romania; dragos.utu@upt.ro (I.-D.U.); viorel.serban@upt.ro (V.-A.Ș.); 2 Technical Sciences Academy of Romania, B-dul Dacia 26, 030167 Bucuresti, Romania

**Keywords:** tribology, coefficient of friction, wear resistance, EPDM, thermoplastic vulcanizate, PA66GF30, pin-on-disk test, material degradation

## Abstract

This study evaluates the tribological behavior of two elastomeric sealing materials—EPDM and TPV—sliding against 30 wt.% glass fiber-reinforced polyamide 66 (PA66GF30), a composite widely used in structural and guiding components. The application context is low-leakage valve systems in polymer electrolyte membrane fuel cells (PEMFCs), particularly on the cathodic (air) side, where dry contact and low-friction sealing are critical. Pin-on-disk tests were conducted under three normal loads (1, 3, and 6 N) and sliding speeds of approximately 0.05, 0.10, and 0.15 m/s (92, 183, and 286 RPM). The coefficient of friction (CoF), mass loss, and wear morphology were analyzed. TPV generally exhibited lower and more stable friction than EPDM, with CoF values exceeding 1.0 at 1 N but falling within 0.32–0.52 under typical operating conditions (≥3 N). EPDM reached a maximum mass loss of 0.060%, while TPV remained below 0.022%. Microscopy revealed more severe wear features in EPDM, including tearing and abrasive deformation, whereas TPV surfaces displayed smoother, more uniform wear consistent with its dual-phase morphology. These findings support the selection of TPV over EPDM in dry-contact sealing interfaces involving composite counterfaces in PEMFC systems.

## 1. Introduction

In recent years, significant advances have been made in the development of wear-resistant materials for demanding dry-contact applications, particularly in the energy and automotive sectors. Zhai et al. [1] emphasized the importance of interface design and phase morphology in tribological performance, highlighting how composite and coated polymeric systems can be tailored for enhanced durability. While tribological properties of EPDM and TPV have been investigated separately, comparative studies involving their direct contact with glass fiber-reinforced polyamide 66 (PA66GF30)—a structural polymer commonly used in valve bodies and guidance systems—remain limited. Previous investigations tend to focus on metal–rubber pairs or frictional behavior under lubrication [2,3]. The tribological response of PA66GF30 in dry conditions, especially as a counterface in fuel-cell sealing components, lacks a systematic comparison across elastomer types.

To the authors’ knowledge, no prior study has systematically compared TPV and silicone-coated EPDM under dry sliding conditions against PA66GF30, despite their widespread use in sealed composite interfaces.

Studies have shown that applying silicone coatings to elastomer surfaces can dramatically reduce interfacial friction and improve contact stability under dynamic loading. For instance, Lee and Kim [4] demonstrated that microstructured silicone-coated rubber exhibited over 70% lower CoF compared to uncoated silicone, owing to reduced surface energy and localized stress absorption. Similarly, Ledig [5] reported that low-friction silicone top coats applied to medical-grade EPDM parts reduced dynamic friction forces by more than 40%, enhancing their performance in valve and sliding-seal applications. In the present work, the EPDM used is similarly treated with a silicone-based surface layer, aiming to replicate these friction-reducing effects and improve wear behavior under dry contact with PA66GF30. This coating plays a key role in defining interfacial shear behavior, and its influence is considered in the interpretation of wear track morphology and material response.

The present study addresses this gap by offering the first direct comparison between TPV and silicone-coated EPDM elastomers in sliding contact with PA66GF30, simulating the operational behavior of low-leakage sealing systems used in PEMFC cathodic-side components. These seals operate under modest mechanical loads (1–6 N) and low to intermediate sliding velocities (0.05–0.15 m/s), without the benefit of liquid lubrication. The silicone surface layer in EPDM is intended to reduce surface energy and improve wear response, while TPV’s dual-phase morphology inherently enhances stress distribution and damage resistance. By evaluating both materials under matched test conditions, this study advances the understanding of elastomer–composite tribo-pairs in real-world sealing environments.

Elastomeric materials, including ethylene propylene diene monomer (EPDM) and thermoplastic vulcanizates (TPV), are widely used in the automobile sector for dynamic sealing applications, gaskets, and vibration dampers. Their tribological performance—specifically, the balance between low friction, high wear resistance, and structural integrity—is critical to system longevity [6,7].

Comprehending the tribological behavior of these materials under varying loads and velocities in conjunction with engineering polymers like polyamide 66 reinforced with 30% glass fiber (PA66GF30) is essential for improving material selection and design [8,9].

Prior research has investigated the tribological performance of EPDM under dry sliding instances and diverse testing settings. Karger-Kocsis et al. [10] indicated that incorporating carbon black into EPDM reduced the coefficient of friction and enhanced wear resistance, with friction coefficients varying from 0.65 to 0.45 based on filler content. Felhös et al. [11] reported that peroxide-cured EPDM with increased filler content had reduced wear rates but escalated surface roughness during testing. Sohail Khan et al. [12] found that EPDM filled with PTFE micro powder exhibited substantial decreases in wear rates and friction coefficients, with values as low as 0.2 under certain conditions.

Thermoplastic vulcanizate materials have also been studied for their tribological performance. Karger-Kocsis et al. [13] observed that TPV materials showed stable friction coefficients in the range of 0.3 to 0.4 under unlubricated conditions against steel, with minimal material transfer and well-defined wear tracks, while Karger-Kocsis et al. [14] demonstrated that carbon nanofiber reinforcement may significantly enhance wear resistance and lower friction, attaining friction coefficients as low as 0.25 and exhibiting enhanced fretting wear resistance with smoother wear surfaces and less debris generation.

The interaction between PA66GF30 and elastomeric materials adds more complications. The tribological response of polymeric materials is known to be strongly influenced by their surface structure, molecular morphology, and modification history. For instance, Bartošová et al. [15] investigated the impact of accelerated electron irradiation on PET, PTFE, and PE2000C, revealing that changes in surface roughness directly influenced the coefficient of friction (CoF). Their study demonstrated that controlled surface modification could reduce CoF values as low as 0.042, although excessive radiation led to degradation and increased friction. These findings underscore the sensitivity of polymer–polymer interfaces to morphological and surface changes. In this context, the dual-phase morphology of TPV and the silicone-coated surface of EPDM may play a similar role in defining friction stability, wear resistance, and energy dissipation at the contact interface.

The current study builds on this understanding by comparing these two elastomeric systems against a fiber-reinforced polymer (PA66GF30) under dry conditions relevant to PEMFC sealing applications. Glass fiber-reinforced polyamide 66 is extensively used in automotive and industrial sectors because of its superior mechanical strength, stiffness, and abrasion resistance. Nonetheless, it is also abrasive when in contact with softer materials such as EPDM or TPV. The comparative performance of EPDM and TPV relative to PA66GF30 is not well recorded; nonetheless, prevailing trends suggest that EPDM, characterized by greater elasticity and reduced hardness, may demonstrate increased wear compared to TPV under equivalent circumstances. In contrast, TPV, because of its elevated Shore A hardness and organized shape, is anticipated to endure the abrasive contact with PA66GF30 more effectively [8,16,17,18].

In light of these interactions, it is also crucial to consider the properties of the counterface material—namely, PA66GF30—whose tribological behavior strongly influences the wear dynamics of the contacting elastomer.

To improve the performance of polyamide composites under high mechanical and thermal stress, various reinforcement strategies have been investigated, among which glass fiber (GF) integration has proven especially effective. Short GF reinforcements increase the stiffness, thermal stability, and dimensional stability of PA66, improving its load-bearing capabilities in tribologically intensive applications such as worm gears and automotive sealing systems [19,20]. The literature reports significant enhancements in wear resistance and friction stability for PA66 composites containing 25–33% GF under dry and lubricated conditions [19,21]. For instance, a study comparing neat PA6 with PA6 + 25% GF revealed an increase in PV limit from 0.78 to 1.04 MPa·m/s, showcasing the fiber’s role in distributing stress and limiting thermal softening [19].

In addition to fiber content, the microstructural development during processing critically influences tribological behavior. Studies have shown that slowly cooled PA66 composites develop a higher fraction of α-phase crystallites and thicker lamellae, both of which enhance mechanical integrity and reduce wear. Cartledge and Baillie observed a 28% improvement in wear resistance and a 14% decrease in friction for slowly cooled GF/PA6 composites compared to their fast-cooled counterparts, attributing these benefits to improved transcrystallinity and fiber–matrix adhesion [22]. These effects are particularly important in polymer–polymer contact configurations, where excessive fiber pull-out or interfacial cracking can trigger rapid surface degradation [22,23].

Under lubricated conditions, particularly when using greases containing zinc carboxylates or similar triboactive additives, PA66GF composites exhibit tribofilm formation at the contact interface. This tribofilm can serve as a barrier to direct asperity contact and reduce both friction and wear. However, the efficacy of tribofilm formation is highly dependent on fiber–matrix bonding. Kunishima et al. demonstrated that CF-reinforced PA66, despite having lower fiber hardness than GF, caused more damage to steel counterparts due to weaker interfacial adhesion and inadequate tribochemical interaction with the grease [20]. These findings highlight the need for optimized fiber–matrix bonding to minimize wear [20,21,22,23,24].

This research aims to comprehensively examine the friction and wear characteristics of low-friction EPDM and TPV materials when tested against PA66GF30 using a pin-on-disk apparatus. The tests were conducted under varied loads (1 N, 3 N, 6 N) and rotating speeds (92, 183, and 286 RPM), emulating various operational circumstances. This study intends to investigate material degradation processes using measurements of the coefficient of friction (CoF), mass loss assessment, and wear track analysis using digital microscopy. The findings provide guidelines for material selection in automobile sealing applications involving elastomeric components in sliding contact with fiber-reinforced polymers.

## 2. Materials and Methods

### 2.1. Materials

The elastomeric materials used in this study were low-friction ethylene propylene diene monomer (EPDM, Uchiyama Europe GmbH, Düsseldorf, Germany) and thermoplastic vulcanizate (TPV, Santoprene™ TPV, Celanese Corporation, Newport, Wales, UK). The EPDM material is characterized by a Shore A hardness of 62, a density of 1.06 g/cm^3^, and excellent flexibility and ozone resistance, making it suitable for automotive sealing applications. The TPV has a Shore A hardness of 80–87, a density of 0.96 g/cm^3^, and notable properties including enhanced dimensional stability, good tear resistance, and excellent weather resistance. Table 1 highlights the most relevant properties of the studied materials.

Both elastomers were chosen in low-friction formulations to reduce energy losses and mitigate heat production during dynamic operations. Polyamide 66, augmented with 30% glass fibers (PA66GF30, supplied by RGPBALLS S.r.l., Cinisello Balsamo, Italy), was used for the stationary ball element in the pin-on-disk testing apparatus. This material was selected for its exceptional stiffness, wear resistance, and structural integrity under stress. Its interaction with elastomeric components replicates authentic automobile conditions, including those present in sliding seals and gasket contacts.

All tribological tests were performed under dry sliding conditions at 23 °C. A single test was conducted for each load–speed combination, with two conditions repeated for verification. Due to this limitation, statistical significance testing was not performed, and the reported differences should be interpreted as indicative trends rather than confirmed by inferential analysis.

### 2.2. Test Equipment and Methodology

**Figure 1:**
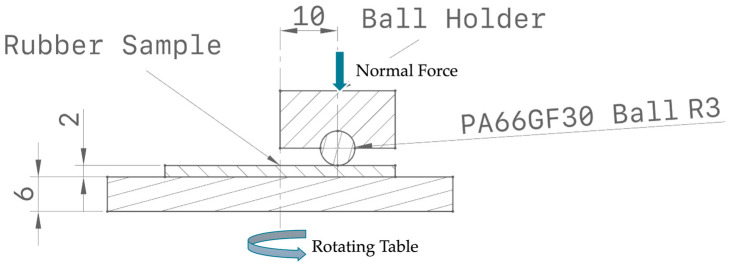
Schematic of the Ball-on-disk Test setup.

Tribological tests were carried out using a TR-20 Micro Pin-on-Disk Tribometer (DUCOM Instruments, Bangalore, India), configured in a ball-on-disk contact mode to simulate elastomer–composite frictional interactions. A schematic representation of the test configuration is shown in Figure 1.

In this setup, the disk consisted of either EPDM or TPV elastomer sheets (2 mm thick), bonded to a metallic support and mounted on the rotating stage. The stationary counterbody was a 6 mm diameter ball made of 30 wt.% glass fiber-reinforced polyamide 66 (PA66GF30). The selected materials and geometry simulate realistic contact conditions in automotive sealing applications.

The normal load was applied vertically using a calibrated dead-weight system mounted on a pivoting lever arm, ensuring repeatable loading conditions. Frictional forces were continuously recorded by a strain gauge sensor installed on the loading arm, allowing real-time calculation of the coefficient of friction (CoF).

The tests were conducted under dry, unlubricated conditions at room temperature (23 °C) for a fixed duration of 30 min per test. Rotational speeds were set at 92, 183, and 286 revolutions per minute (RPM), corresponding to approximate sliding speeds of corresponding to sliding speeds of approximately 0.05, 0.10, and 0.15 m/s, respectively, over a 10 mm circular wear track. The applied normal loads were 1 N, 3 N, and 6 N, covering a realistic range of contact pressures.

Each test was preceded by a surface cleaning step using isopropanol to remove surface contaminants. After testing, the rubber specimens were carefully removed and weighed using a precision analytical balance (ASB-60-220-C2-V2, MRC Ltd., Holon, Israel), with a readability of 0.00001 g for low-mass measurements.

Wear track morphology was examined using a Leica DMS1000 digital microscope (Leica Microsystems, Wetzlar, Germany). Images were captured under uniform lighting and magnification, and wear track dimensions were measured using integrated software tools. The inner and outer diameters of the wear scars were used to calculate the worn surface area, and surface features such as tearing, polishing, and debris accumulation were analyzed. The measurement uncertainty associated with the digital microscopy wear area evaluation was estimated at ±3%, based on repeated measurements of selected tracks and the resolution limits of the Leica DMS1000 system.

## 3. Results

The tribological performance of EPDM and TPV against PA66GF30 was evaluated through a matrix of normal loads (1 N, 3 N, and 6 N) and sliding speeds (92, 183, and 286 RPM). The main findings are presented in terms of coefficient of friction (CoF), mass loss, wear area, and surface morphology.

### 3.1. CoF vs. Time Results

**Figure 2:**
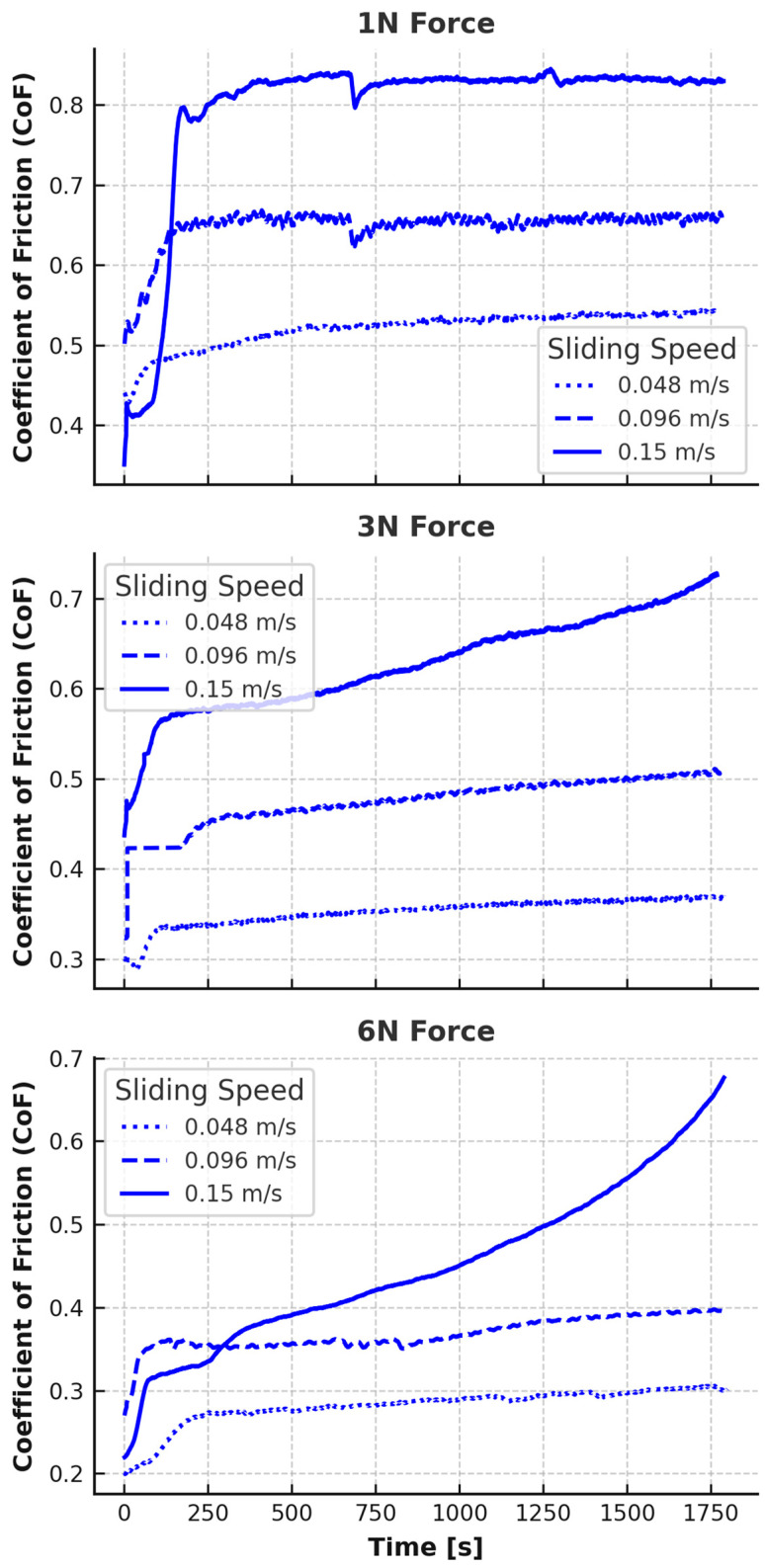
CoF vs. Time for EPDM, grouped by force.

**Figure 3:**
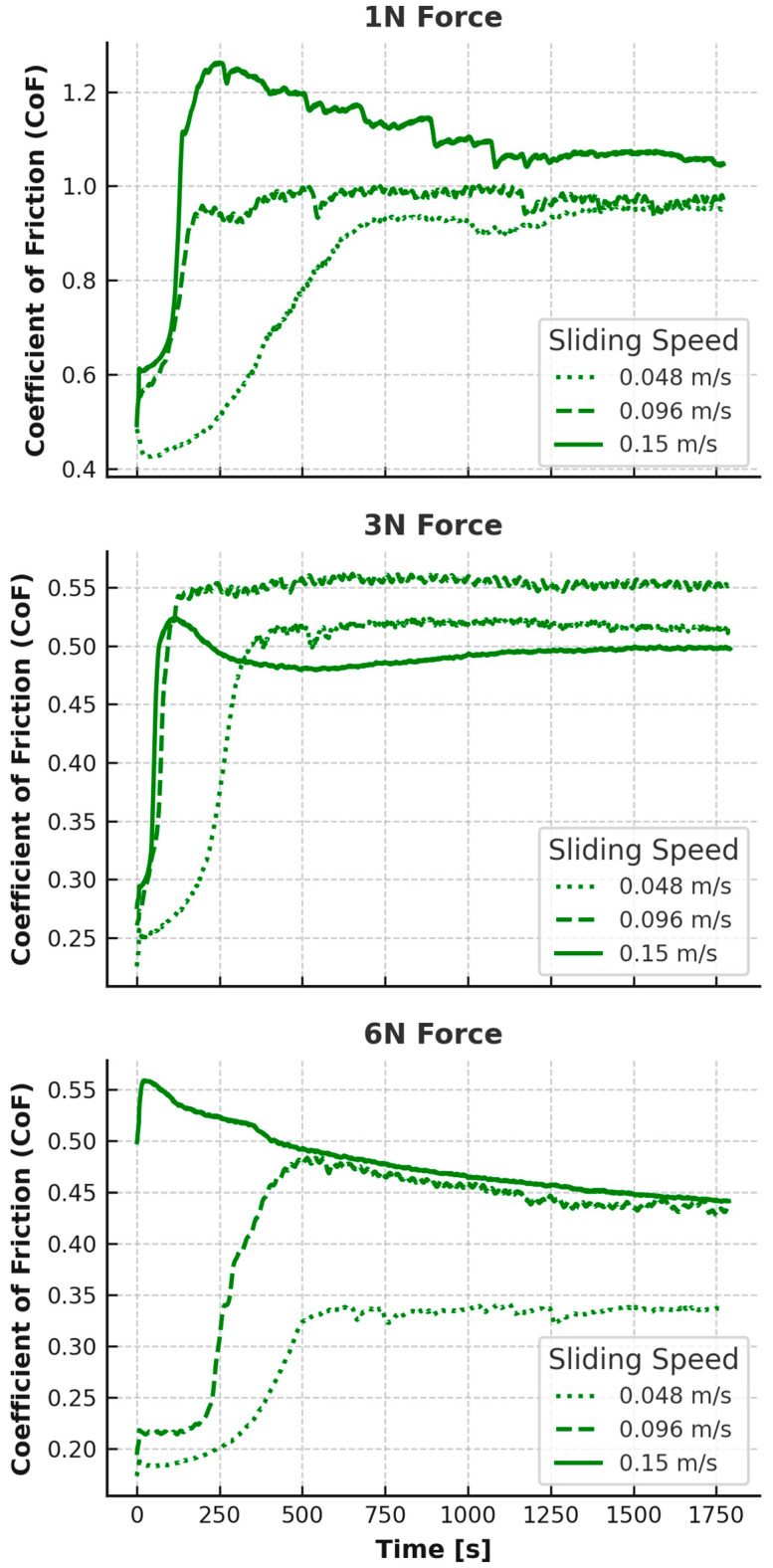
CoF vs. Time for TPV, grouped by force.

The coefficient of friction (CoF) as a function of time for both EPDM and TPV was recorded under varying normal loads (1 N, 3 N, and 6 N) and rotational speeds (0.048, 0.096, and 0.15 m/s). The smoothed CoF curves are presented in Figure 2 and Figure 3.

Each curve features an initial transient phase, typically within the first 150–250 s, followed by a quasi-steady sliding regime. Distinct differences were observed between EPDM and TPV in both absolute CoF values and the shape of their time-dependent responses.

At the lowest test load (1 N), EPDM displayed moderate initial friction values that rapidly increased with RPM. At 0.048 m/s, CoF stabilized around 0.54. At 0.096 m/s and 0.15 m/s, the steady-state CoF increased to approximately 0.67 and 0.83, respectively. The friction rise at higher RPMs indicates increased interfacial shear due to greater velocity and potential thermal softening at the rubber–composite interface. The relatively low initial values at 0.048 m/s suggest a dominant adhesive interaction, while the steep rise with speed reflects a shift toward deformation-driven and fatigue-assisted frictional mechanisms.

For TPV at 1 N, an opposite trend was observed: friction was high at low speed (stabilizing above 0.9 at 0.048 m/s), and even higher at 0.096 m/s and 0.15 m/s, exceeding 1.0 in a transient manner, but stabilizing closer to ~0.9–1.0 at 0.15 m/s. These high values at low load suggest a strong adhesive interface due to insufficient contact pressure to compress TPV’s surface texture. The thermoplastic matrix likely increased surface stiffness under low load, resulting in elevated real contact area and interfacial shear.

At the intermediate load (3 N), EPDM’s CoF stabilized between 0.44 (0.048 m/s) and 0.67 (0.15 m/s), with a monotonic increase in friction and time-dependent instability at high speeds. The increasing slope of the CoF curves at 0.15 m/s indicates that wear or surface damage may be progressively contributing to contact evolution. TPV, on the other hand, showed highly stable curves at all speeds, with CoF stabilizing around 0.41 (0.048 m/s), 0.47 (0.096 m/s), and 0.50 (0.15 m/s). The relatively flat profile suggests minimal microstructural change during sliding and effective stress redistribution through the TPV’s hybrid phase structure.

At the highest load of 6 N, EPDM continued its upward trend. While the initial values remained modest (e.g., ~0.28 at 0.048 m/s), CoF rose steadily throughout the test at 0.096 m/s and 0.15 m/s, ultimately reaching ~0.50 and ~0.68, respectively, after 30 min. This slow, linear rise over time underlines the material’s increasing susceptibility to fatigue and surface disruption, especially at high shear velocities. TPV at 6 N showed the opposite behavior: a short transient followed by steady-state friction of ~0.36 (0.048 m/s), ~0.41 (0.096 m/s), and ~0.45 (0.15 m/s), with a slight decreasing trend toward the end of the test in some cases, indicating stabilization or partial smoothing of the contact interface.

Overall, EPDM displayed higher and more time-variant CoF values, particularly under high-speed and high-load conditions, while TPV maintained lower, more stable friction across all test parameters. These differences are rooted in material morphology: EPDM’s homogeneous, highly elastic structure leads to continuous surface adaptation, while TPV’s dual-phase morphology enhances dimensional stability and limits frictional energy dissipation.

**Figure 4:**
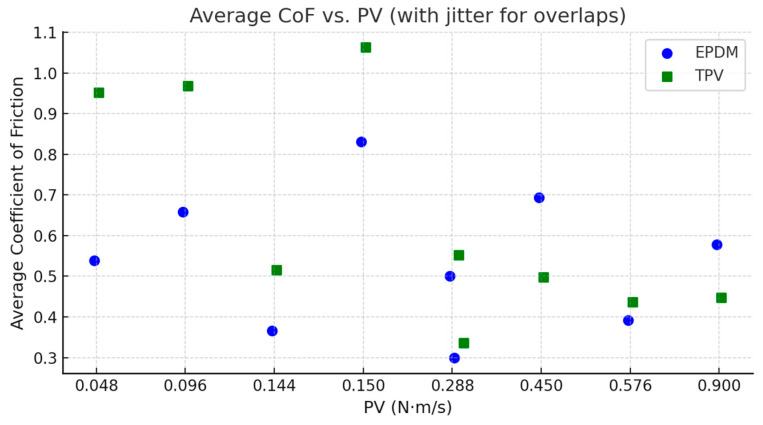
Average coefficient of friction vs. PV (Pressure × Velocity) for EPDM and TPV. Slight horizontal jitter was applied to overlapping PV values to distinguish different test conditions with identical PV.

Figure 4 presents the relationship between the calculated PV (Pressure × Velocity) parameter and the average coefficient of friction for both EPDM and TPV. A slight horizontal jitter was applied to data points corresponding to repeated PV values arising from different force–velocity combinations (e.g., 3 N at 0.096 m/s and 6 N at 0.048 m/s) to enhance visual clarity. The results show that TPV exhibits a lower average coefficient of friction across the PV spectrum, particularly at higher PV levels, suggesting better interfacial stability and reduced shear resistance under increased contact stress.

### 3.2. Mass Loss

Mass loss measurements were taken by recording the initial weight of each elastomer sample before testing and the final weight after testing, providing quantitative data on material wear. The results indicate a clear relationship between applied force, rotational speed, and mass loss.

The mass loss (∆*m*) was calculated using the following formula:

(1) $$\Delta m\left(\%\right)=\left(\left(m_{initial}-m_{final}\right)/m_{initial}\right)*100,$$

where $m_{initial}$ is the mass of the sample before testing and $m_{final}\;$ is the mass after testing.

**Figure 5:**
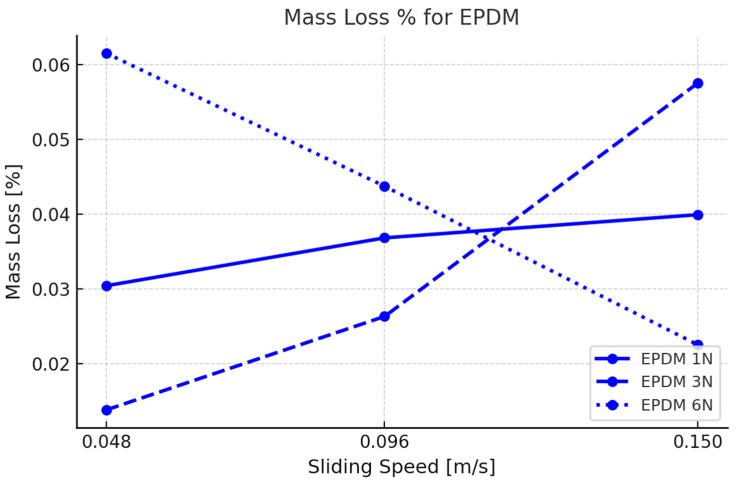
Mass Loss% vs. sliding speed [m/s] for EPDM across the different forces.

For EPDM (Figure 5), the mass loss increased consistently with both normal load and rotational speed. At the lowest test condition (1 N, 0.048 m/s), EPDM exhibited a baseline mass loss of approximately 0.016%. As load and speed increased, wear intensified. At 3 N, the mass loss ranged from approximately 0.034% at 0.048 m/s to 0.045% at 0.15 m/s, reflecting a clear positive correlation with sliding velocity. At the most aggressive setting (6 N, 0.15 m/s), EPDM reached its maximum wear value of approximately 0.060%, indicating significant material degradation under combined mechanical and thermal stress. This behavior is indicative of EPDM’s vulnerability to fatigue and plowing wear mechanisms, particularly when tested against a hard, fiber-reinforced PA66GF30 surface.

**Figure 6:**
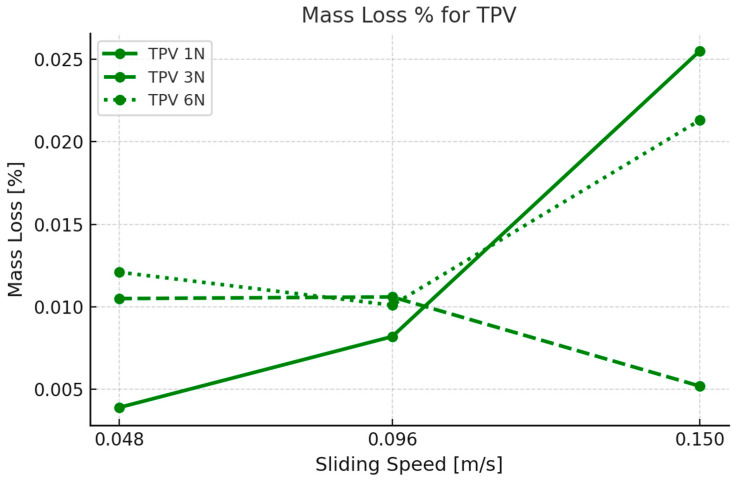
Mass Loss% vs. sliding speed [m/s] for TPV across the different forces.

In contrast, TPV (Figure 6) demonstrated lower and more nuanced mass loss behavior. At 1 N, the mass loss increased with speed, starting from ~0.004% at 0.048 m/s and reaching ~0.012% at 0.15 m/s. However, the rate of increase was not as steep as with EPDM. For 3 N, TPV showed minimal variation, maintaining a fairly constant mass loss value around 0.010–0.011% across all speeds, suggesting that moderate loading enables TPV to maintain consistent wear resistance regardless of sliding velocity. At the highest load of 6 N, TPV followed a more expected trend, with mass loss rising from ~0.009% at 0.048 m/s to ~0.022% at 0.15 m/s, again confirming increased material removal under more intense operating conditions.

The results suggest that TPV may be better suited for dry-contact applications, pending further validation. Even at 6 N and 0.15 m/s, TPV’s mass loss remained significantly below that of EPDM, with values around 0.022% versus 0.060%, respectively. This threefold reduction in wear under the same test conditions highlights the superior durability of TPV’s hybrid thermoplastic-elastomeric structure. Additionally, TPV’s less aggressive mass loss increases with load and speed, indicating a more stable contact interface and better resistance to shear and fatigue damage during prolonged sliding.

Notably, EPDM displayed a nonlinear increase in mass loss with increasing test severity, suggesting that beyond a certain threshold, the material enters an unstable wear regime dominated by surface tearing and delamination. In TPV, the rate of mass loss was more gradual and load-dependent, with minimal sensitivity to speed at intermediate forces. This behavior likely stems from TPV’s ability to distribute interfacial stress more evenly, minimizing localized failure.

**Figure 7:**
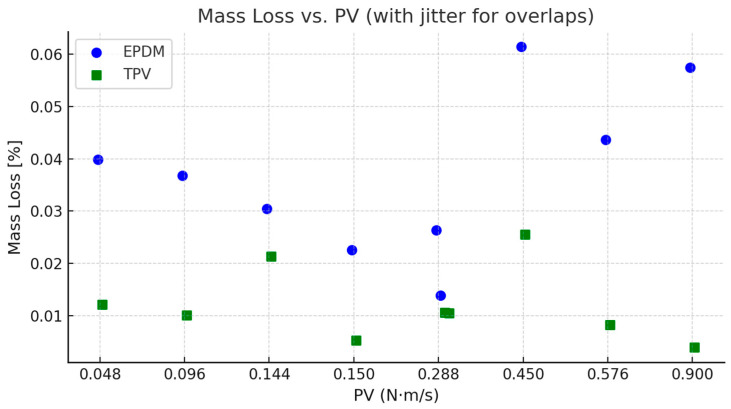
Mass loss [%] vs. PV (Pressure × Velocity) for EPDM and TPV. Horizontal jitter was applied to distinguish data points with identical PV values resulting from different force–speed combinations.

Figure 7 shows the variation in mass loss with respect to the PV (Pressure × Velocity) values for EPDM and TPV. To accommodate overlapping PV values from different test conditions (e.g., 3 N at 0.096 m/s and 6 N at 0.048 m/s), a small horizontal jitter was introduced to ensure all data points remain visually distinct. TPV demonstrates a significantly lower mass loss across the tested PV range, reinforcing its superior wear resistance and mechanical integrity under elevated contact pressure and sliding speeds.

These findings reinforce the conclusion that TPV is better suited for tribological applications involving contact with fiber-reinforced composites such as PA66GF30, especially under high-load, high-speed conditions where wear resistance is critical.

While the mass loss differences between EPDM and TPV appear consistent with the observed wear track morphology, the single-replicate design limits conclusions on statistical significance. The observed differences should be interpreted with this consideration in mind.

### 3.3. Wear Area and Observed Damage

**Figure 8:**
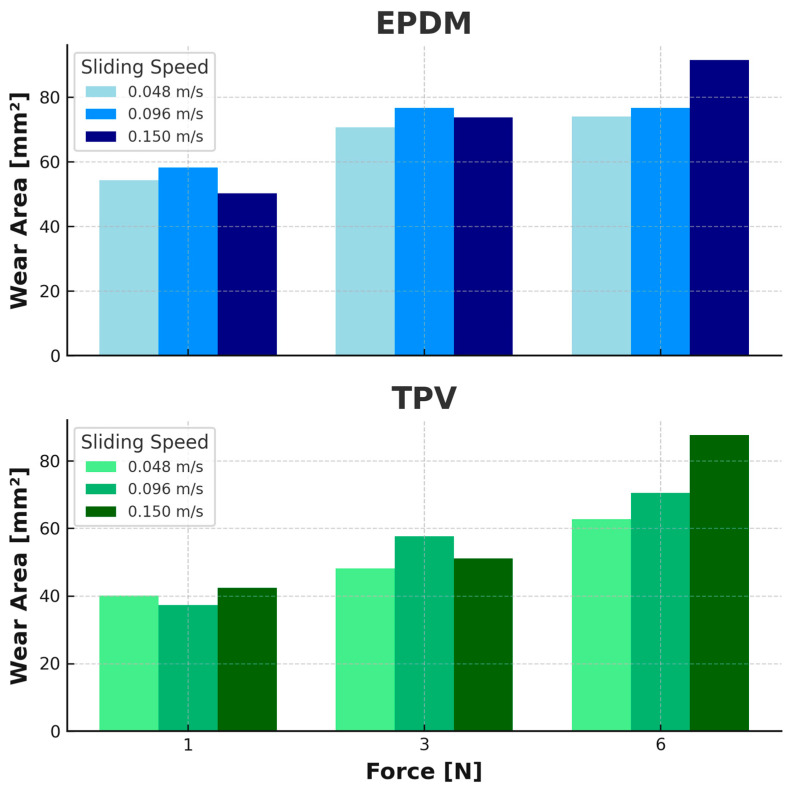
Wear area by Force and rotational speed for EPDM (**Top**) and TPV (**Bottom**).

The wear areas were calculated using calibrated optical measurements obtained via the Leica DMS1000 digital microscope. For each test, the inner and outer diameters of the annular wear tracks were recorded, and the worn surface area (in mm^2^) was computed using the standard formula for ring-shaped geometries (2). The results are shown in Figure 8.

(2) $$A=\pi *\left({\left(D_{outer}/2\right)}^{2}-{\left(D_{inner}/2\right)}^{2}\right).$$

EPDM exhibited a progressive increase in wear area with both normal load and sliding speed. At 1 N, wear areas ranged from approximately 50 mm^2^ at 0.048 m/s to 58 mm^2^ at 0.096 m/s, followed by a slight decrease at 0.15 m/s. At 3 N, wear areas increased from ~70 mm^2^ at 0.048 m/s to a peak of ~77 mm^2^ at 0.096 m/s, and slightly decreased at 0.15 m/s. For 6 N, the wear area increased steadily from ~74 mm^2^ to ~91 mm^2^ across the same speed range. In contrast, TPV exhibited significantly lower wear areas at 1 N and 3 N across all speeds. Only at 6 N and 0.15 m/s did TPV approach EPDM wear levels, recording 87.7 mm^2^ vs. 91.6 mm^2^ for EPDM.

**Figure 9:**
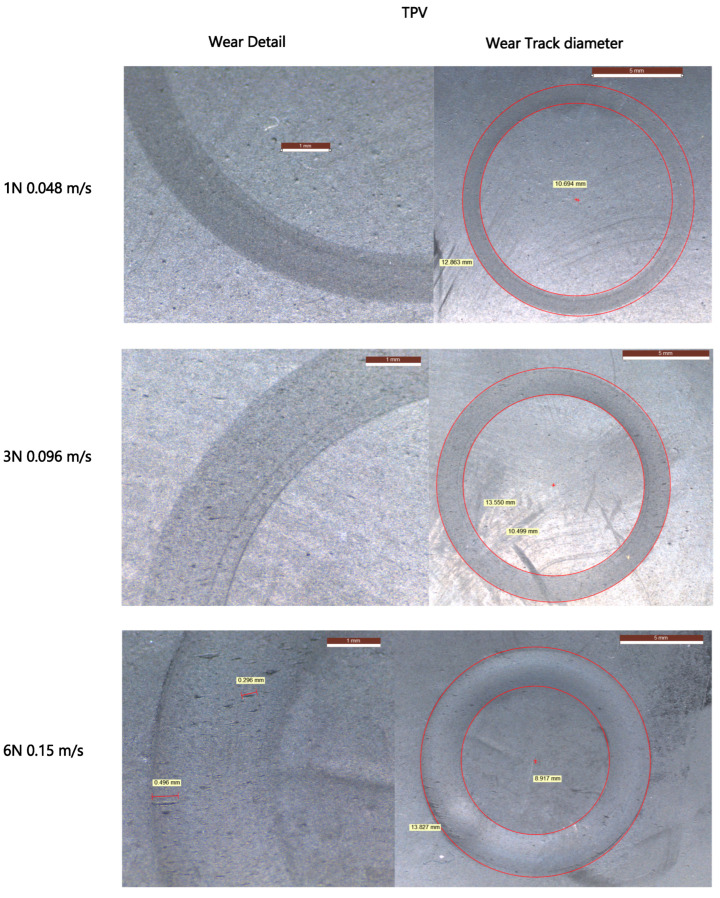
Digital microscopy images of localized wear defects (**left**) and corresponding full wear tracks (**right**) for EPDM samples tested under increasing normal loads and sliding speeds: 1 N at 0.048 m/s (**top row**), 3 N at 0.096 m/s (**middle row**), and 6 N at 0.15 m/s (**bottom row**).

**Figure 10:**
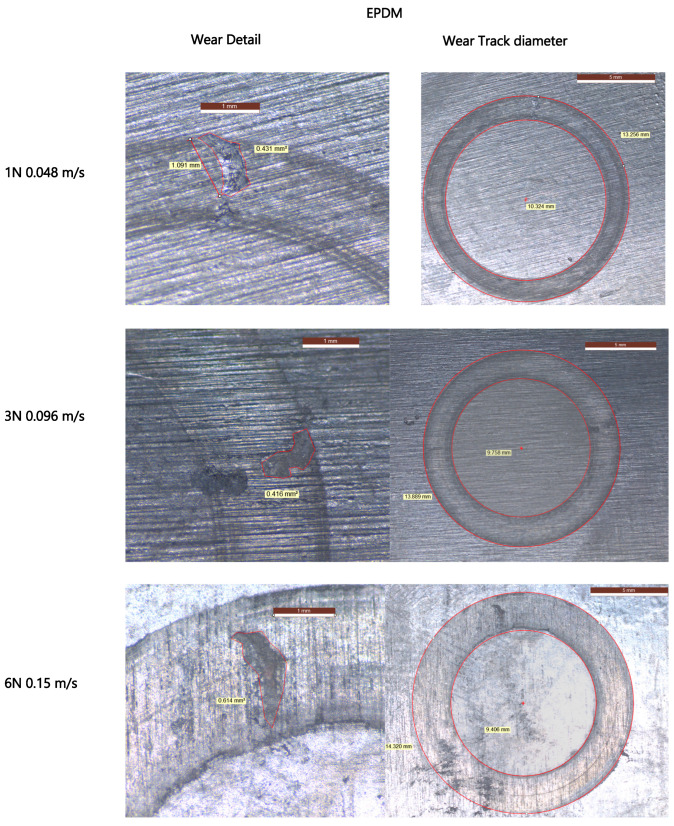
Digital microscopy images of TPV wear tracks (**left**: close-up, **right**: full track) under increasing normal loads and sliding speeds: 1 N at 0.048m/s (**top row**), 3 N at 0.096 m/s (**middle row**), and 6 N at 0.15 m/s (**bottom row**), in contact with PA66GF30.

The wear tracks observed in Figure 9 and Figure 10 show visible morphological differences between the two materials. EPDM surfaces reveal wider wear paths with irregular track boundaries and accumulated wear debris, particularly under higher loads and speeds. TPV tracks appear narrower and smoother, with continuous and uniform wear paths even under increased test severity. These visual differences suggest a more stable and resilient wear behavior in TPV.

Although no quantitative profilometry was conducted, digital microscopy revealed clear topographical evolution. EPDM tracks exhibited increased roughness and edge tearing with higher PV, while TPV surfaces showed evidence of progressive smoothing, consistent with tribo-layer formation and wear stability.

To estimate the total volume of material loss, wear volume was calculated by multiplying the measured cross-sectional wear area by the circumferential path length of the track. The following equations were used:

(3) $$D_{\text{mean}}=\left(D_{\text{outer}}+D_{\text{inner}}\right)/2,$$

(4) $$C=\pi \times D_{\text{mean}},$$

(5) V=A×C

- *C* is the circumference of the wear track (mm),
- *A* is the measured wear area (mm^2^),
- *V* is the calculated wear volume (mm^3^).

**Figure 11:**
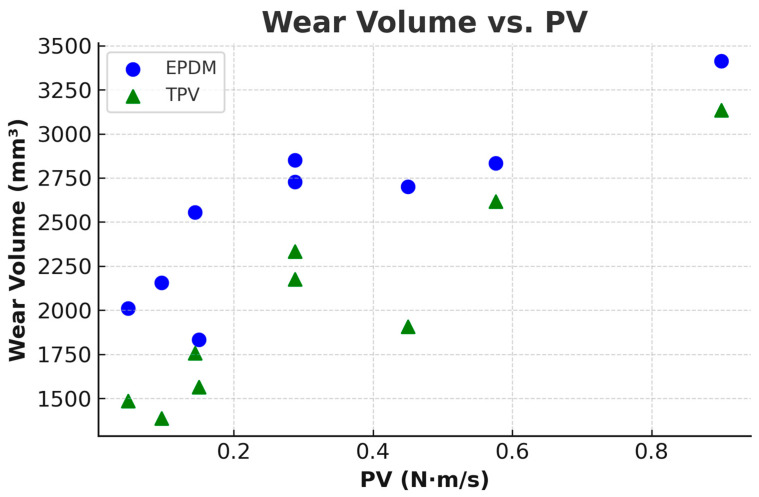
Wear volume of EPDM and TPV samples as a function of the PV parameter.

The resulting wear volumes are plotted against the PV parameter (pressure × velocity) in Figure 11. EPDM exhibits increasing wear volume with PV, reaching a maximum of approximately 3412 mm^3^ at 0.9 N·m/s. TPV exhibited reduced wear volumes across most PV values, remaining below 2200 mm^3^ except at the highest test condition (0.9 N·m/s), where both materials reached comparable values (~3412 mm^3^). Only at the highest PV do both materials reach comparable wear volumes, indicating that under extreme loading, both experience substantial material degradation.

## 4. Discussions

A known limitation of the pin-on-disk configuration is the radial variation in sliding velocity across the contact path, which can influence local wear and friction. Kohutiar et al. [25] demonstrated that tribological results can differ significantly based on the movement geometry, with circular tests showing up to 147% higher wear compared to linear motion using the same material pairs and test parameters. In our study, this effect was mitigated by using a fixed track radius of 5 mm, corresponding to a wear path diameter of approximately 10 mm, and a narrow contact band (~3–4 mm width), which limited sliding speed variation across the wear zone. Although minor gradients remain, their effect on average CoF and wear volume is likely minimal. The wear morphologies observed—especially the coexistence of delamination and abrasive features—are consistent with such mixed-motion conditions as described by Kohutiar et al. [25].

### 4.1. EPDM Performance Against PA66GF30

EPDM exhibited distinct tribological behavior depending on the applied load and rotational speed. At 1 N, the coefficient of friction (CoF) ranged from approximately 0.50 (0.048 m/s) to 0.82 (0.15 m/s). At 3 N, the CoF ranged from 0.34 to 0.65, and at 6 N it peaked above 0.68 for 0.15 m/s, showing a strong dependency on both load and speed. Notably, the friction response increased significantly with force and sliding speed, suggesting enhanced viscoelastic deformation and adhesion at the contact interface.

The mass loss for EPDM ranged from 0.016% (1 N, 0.048 m/s) to 0.060% (6 N, 0.15 m/s). This progressive increase in wear with higher mechanical inputs indicates the material’s limited resistance to fatigue and plowing effects when in contact with the rough, fiber-filled surface of PA66GF30. This trend suggests EPDM’s susceptibility to mechanical degradation under stress, which agrees with prior studies on unreinforced EPDM [10,11].

Digital microscopy revealed surface tearing, wrinkling, and pronounced wear scars, especially under higher RPMs, confirming fatigue-driven abrasive mechanisms. These patterns reflect insufficient structural reinforcement in EPDM to cope with the hard, composite counterface, where glass fiber ends promote micro-cutting and plowing actions [26].

### 4.2. TPV Performance Against PA66GF30

TPV outperformed EPDM across all tribological parameters. CoF values were significantly lower: at 1 N, CoF reached a maximum of ~1.15 initially but stabilized around 1.0 (0.15 m/s) and ~0.9 (0.096 m/s). At 3 N and 6 N, friction values ranged between 0.25–0.55, clearly lower than EPDM under comparable conditions.

The mass loss of TPV ranged from ~0.004% (1 N, 0.048 m/s) to ~0.022% (6 N, 0.15 m/s)—up to three times lower than EPDM. Even under the most aggressive conditions, TPV maintained a relatively modest wear response, indicating greater structural resilience. This behavior is attributed to TPV’s dual-phase morphology, where the thermoplastic matrix offers rigidity, and the crosslinked rubber phase ensures elastic recovery during repeated contact cycles [27].

Microscopic examination supported these findings: TPV surfaces presented narrower, more regular wear tracks, reduced delamination, and an absence of large-scale surface rupture. This smoother wear morphology suggests more stable tribo-film development and reduced fiber penetration depth.

### 4.3. Comparative Insights and the Role of PA66GF30

The tribological behavior observed in this study is consistent with prior work on rubber–polymer interfaces and filler-enhanced elastomers. In particular, the superior wear resistance of TPV can be attributed to its microphase-separated morphology, where fine rubber domains dispersed in a thermoplastic matrix serve to arrest crack propagation and reduce surface damage under shear. This architecture promotes more uniform deformation and stress redistribution during sliding, as confirmed by Karger-Kocsis et al. in TPV–metal contact systems [10].

For EPDM, increased wear and tearing are in line with literature reports that attribute such behavior to abrasive fiber interactions and the absence of internal morphological barriers to stress concentration. Felhös et al. demonstrated that wear in carbon-black-filled EPDM increases with filler content due to stiffness gradients and frictional heating [11]. Similarly, Khan et al. and Martínez et al. reported unstable friction in EPDM-polyamide contacts at dry interfaces, especially at higher pressures and speeds, where adhesive-to-abrasive wear transitions are more likely [27,28]. These findings are supported by De Sousa et al. [29], who observed comparable frictional instabilities and surface roughening in rubber–polyamide contacts under dry sliding conditions.

TPV’s ability to maintain lower CoF and wear volumes in this study aligns with findings from fretting and reciprocating sliding experiments on Santoprene-type TPVs, which also exhibited smoother wear scars and limited crack initiation. The positive role of structured or silicone-modified surfaces in reducing friction, as shown by Lee & Kim (2023) [4] and Ledig (2010) [5], provides further theoretical support for the frictional improvement observed in the silicone-coated EPDM variant tested here.

Additionally, microscopic fiber protrusion or fiber–matrix debonding in PA66GF30 may exacerbate localized cutting or plowing of the elastomeric surface. This mechanism likely contributes to the observed transition from primarily adhesive wear at low PV to a more abrasive regime, especially for EPDM, which lacks a rigid support matrix to resist such interactions [23,24].

Finally, PV-related wear escalation in EPDM and TPV echoes trends seen in PTFE-modified composites, where fiber orientation, load transfer, and temperature effects combine to affect wear depth and scar geometry under dynamic sliding.

### 4.4. Limitations and Future Work Perspectives

Although this study provides detailed tribological insights into the dry contact behavior of EPDM and TPV against PA66GF30, several limitations should be acknowledged. First, only single-replicate tests were performed for most conditions due to material and equipment constraints, which prevents full statistical validation of the observed trends. Second, the microstructural characterization was limited to optical digital microscopy. While this technique enabled qualitative comparison of wear scar morphology, it does not provide sufficient resolution to analyze micro-crack propagation, fiber pull-out, or subsurface deformation mechanisms. Future work will incorporate scanning electron microscopy (SEM) and 3D profilometry to quantitatively assess surface topography, localized damage, and the evolution of roughness under varying tribological loads. Additionally, follow-up investigations will include counterface wear evaluation, thermally accelerated tests, and potential effects of lubricated environments to better simulate real-world conditions in PEMFC and automotive sealing systems.

## 5. Conclusions

The tribological behavior of EPDM and TPV elastomers in dry contact with glass fiber-reinforced PA66 (PA66GF30) was evaluated under varying loads and sliding speeds using a pin-on-disk tribometer. While limited by a predominantly single-replicate test design, the observed trends provide insight into material performance for polymer–polymer sealing interfaces. The following indicative conclusions are drawn:

1. Friction Coefficient Performance:

EPDM generally exhibited higher average coefficients of friction (CoF) than TPV across all test conditions. For example, under 1 N and 0.048 m/s, EPDM reached a mean CoF of ~0.52 compared to TPV’s ~0.45. At 6 N and 0.15 m/s, EPDM demonstrated a CoF of ~0.45, with a constant increase of CoF with time, while TPV stabilized to ~0.45, indicating greater interfacial stability and reduced energy dissipation.

2. Wear Mass Loss Trends:

TPV demonstrated lower mass loss than EPDM, with reductions ranging from ~20% to over 50% depending on the condition. At 3 N and 0.096 m/s, EPDM lost 0.045% of its original mass, while TPV only lost 0.039%. The most severe case, at 6 N and 0.15 m/s, showed both materials converging near ~0.09% mass loss, suggesting a critical degradation threshold at high PV.

3. Wear Area and Surface Morphology:

Digital microscopy revealed that EPDM produced larger wear areas, ranging from 50.3 mm^2^ (1 N, 0.15 m/s) up to 91.6 mm^2^ (6 N, 0.15 m/s). In contrast, TPV wear areas were lower in 8 of 9 conditions, reaching a maximum of 91.6 mm^2^ only at the highest PV. EPDM scars were visibly wider and more irregular, with edge debris and plowing features, while TPV exhibited smoother, more defined tracks with minimal lateral damage.

4. Wear Volume vs. PV:

Wear volume increased with the PV product for both materials, but TPV exhibited more stability. EPDM’s wear volume ranged from 1833 mm^3^ (1 N, 0.15 m/s) to 3412 mm^3^ (6 N, 0.15 m/s), while TPV remained below 2200 mm^3^ in most cases, except at 6 N, where it also reached 3412 mm^3^. This convergence suggests that both materials reach similar wear behavior at extreme contact energy inputs.

5. Material Morphology and Wear Resistance:

TPV’s superior wear resistance is attributed to its dual-phase microstructure: a dispersed rubber phase within a thermoplastic matrix, which enhances crack resistance and stress redistribution. Literature-supported observations confirm that such morphologies reduce tearing and fatigue failure in elastomer–polymer contacts, unlike the homogeneous EPDM matrix.

6. Implications for Sealing and Polymer–Polymer Interfaces:

The results demonstrate that TPV is better suited for dry-contact applications against glass–fiber composites such as PA66GF30, especially under dynamic loads. TPV’s reduced friction (by up to 0.1 absolute) and up to 55% lower wear mass loss at moderate PV conditions make it a more durable candidate for long-term sealing, guiding, or dynamic coupling in fuel cell or automotive subsystems.

## Figures and Tables

**Table 1:** Comparison of Mechanical and Thermal Properties of EPDM and TPV.

Property	LF EPDM	LF TPV
Hardness (Shore A)	62	80–87
Density (g/cm^3^)	1.06	0.96
Tensile Strength (MPa)	19.4	10.4
Elongation at Break (%)	330	530
Compression Set (%)	11 (24 h at 125 °C)	36–52 (22–70 h at 70–125 °C)
Working Temperature (°C)	−40 to +125 (typical)	−60 to +135 (typical continuous use)
Ozone & Weather Resistance	Excellent	Excellent
Typical Uses	Seals, gaskets, vibration dampers, automotive	Seals, bellows, tubing, gaskets, automotive parts
Tg (°C)	−50	−40
Storage Modulus (E′) at 25 °C (MPa)	1.0–3.5 MPa depending on filler	6 MPa

## Data Availability

The original contributions presented in this study are included in the article. Further inquiries can be directed to the corresponding author.
